# Ethanol Intoxication Alleviates the Inflammatory Response of Remote Organs to Experimental Traumatic Brain Injury

**DOI:** 10.3390/ijms21218181

**Published:** 2020-10-31

**Authors:** Baolin Xu, Akila Chandrasekar, Florian olde Heuvel, Maciej Powerski, Aleksander Nowak, Laurens Noack, Jazan Omari, Markus Huber-Lang, Francesco Roselli, Borna Relja

**Affiliations:** 1 Experimental Radiology, Department of Radiology and Nuclear Medicine, Otto von Guericke University, 39120 Magdeburg, Germany; xubaolin325@outlook.com (B.X.); maciej.powerski@med.ovgu.de (M.P.); Aleksander.nowak@med.ovgu.de (A.N.); laurens.noack@st.ovgu.de (L.N.); jazan.omari@med.ovgu.de (J.O.); 2 Department of Neurology, Ulm University, 89081 Ulm, Germany; a.chandrasekar@tu-braunschweig.de (A.C.); florian.oldeheuvel@gmail.com (F.o.H.); 3 Institute of Clinical and Experimental Trauma-Immunology, University Hospital Ulm, Ulm University, 89081 Ulm, Germany; mshuberlang2000@aol.com; 4 German Center for Neurodegenerative Diseases (DZNE)-Ulm, 89081 Ulm, Germany; francesco.roselli@dzne.de

**Keywords:** inflammation, TBI, alcohol, cytokines, lung, liver

## Abstract

Traumatic brain injury (TBI) may cause damage to distant organs. Acute ethanol intoxication (EI) induces complex local and systemic anti-inflammatory effects and influences the early outcomes of traumatized patients. Here, we evaluated its effects on the BI-induced expression of local inflammatory mediators in the trauma-remote organs the lungs and liver. Male mice were exposed to ethanol as a single oral dose (5g·kg^–1^, 32%) before inducing a moderate blunt TBI. Sham groups underwent the same procedures without TBI. Ether 3 or 6h after the TBI, the lung and liver were collected. The gene expression of HMGB1, IL-6, MMP9, IL-1β, and TNF as well as the homogenate protein levels of receptor for advanced glycation end products (RAGE), IL-6, IL-1β, and IL-10 were analyzed. Liver samples were immunohistologically stained for HMGB1. EI decreased the gene expressions of the proinflammatory markers HMGB1, IL-6, and MMP9 in the liver upon TBI. In line with the reduced gene expression, the TBI-induced protein expression of IL-6 in liver tissue homogenates was significantly reduced by EI at 3h after TBI. While the histological HMGB1 expression was enhanced by TBI, the RAGE protein expression in the liver tissue homogenates was diminished after TBI. EI reduced the histological HMGB1 expression and enhanced the hepatic RAGE protein expression at 6h post TBI. With regard to the lungs, EI significantly reduced the gene expressions of HMGB1, IL-6, IL-1β, and TNF upon TBI, without significantly affecting the protein expression levels of inflammatory markers (RAGE, IL-6, IL-1β, and IL-10). At the early stage of TBI-induced inflammation, the gene expression of inflammatory mediators in both the lungs and liver is susceptible to ethanol-induced remote effects. Taken together, EI may alleviate the TBI-induced pro-inflammatory response in the trauma-distant organs, the lungs and liver, via the HMGB1-RAGE axis.

## 1. Introduction

Traumatic brain injury (TBI) is globally the main cause of death and disability and is a growing public health concern among all trauma-related injuries, especially for young adults [1,2]. A European survey study has reported that the rough incidence of TBI ranges from 47/100,000 to 694/100,000 people per year, and that the mortality rates from TBI ranged from 9 to 28/100,000 people per year for all ages and TBI severities [3]. An important risk factor for TBI is binge ethanol intoxication, in particular in the case of accidental falls and bicycle accidents [4]. In a systematic review and meta-analysis, Ding et al. have reported that blood alcohol concentration (BAC) has not been associated with death in patients suffering from TBI, though lower BAC levels were associated with greater odds of death than higher BAC levels [5]. However, Ding et al. included not only the TBI-exclusive population in corresponding research studies in their meta-analysis, because TBI occurs often simultaneously with multi-trauma and isolated TBI itself is rather rare. Due to this and the limited knowledge on the impact of TBI on post-traumatic systemic and remote organ injury and regeneration, the authors correctly stated that their mortality data are actually inconsistent, underlining the need for further data in isolated TBI.

The post-traumatic outcome of patients is markedly affected by inflammatory processes set in motion at the levels of the brain parenchyma by TBI but then systemically spreading to distant organs such as the lungs, liver, heart, gastrointestinal tract, kidney, spleen, and bone [6,7], where inflammation may cause life-threatening organ dysfunction. For example, besides direct brain injury, TBI can induce indirect complications, such as TBI-induced acute lung injury (ALI) or acute respiratory distress syndrome (ARDS) [8]. Thus, the outcome of patients suffering from TBI is markedly influenced not only by local but also inflammatory processes unfolding throughout the body [9]. Immediately after brain injury, the release of the endogenous danger-associated molecular pattern (DAMP) and the activation of its corresponding receptors induce astrocytic and microglial responses, including the generation of cytokines and chemokines [10]. High-mobility group box (HMGB)-1, as a classical DAMP, is activated and released into extracellular space upon TBI [11,12]. Interestingly, HMGB1 also initiates the local inflammatory response in the lungs via binding the receptor for advanced glycation end products (RAGE), which is highly expressed in lung epithelial cells after TBI [8]. Adjacent to their increase in cerebrospinal fluid, TBI upregulates the levels of circulating proinflammatory cytokines as well, including interleukin (IL)-6 or tumor necrosis factor alpha (TNF-α) [9]. After TBI, the inflammasome proteins encapsulated by extracellular vesicles are systemically released from the brain, thus targeting the lungs and causing ALI via a neural-respiratory-inflammasome axis [13].

Concerning the liver, TBI can exert a direct modulation of the synthesis of acute phase proteins (e.g., serum amyloid A, C reactive protein, or haptoglobin) by hepatocytes [6,14,15]. The liver is also centrally involved in the neuro-innate immune response after trauma and TBI [16]. In situ, acute ethanol intake significantly reduces the levels of IL-1β and TNF-α in the injured cortex 4h after TBI, accompanied with an augmentation of the serum corticosterone level in a dose-dependent manner [17]. In a porcine fluid-percussion model of TBI, ethanol intoxication caused abnormal changes in hemodynamics (e.g., lower mean arterial blood pressure, lower cerebral perfusion pressure, and lower cerebral blood flow) and respiratory function (e.g., lower ventilation, lower hypercapnic response sensitivity, higher PaCO_2_, and longer postinjury apneas), which may have fateful outcomes after the TBI [18,19]. As demonstrated in numerous studies, alcohol influences systemic inflammatory mediators [20] as well as outcomes in experimental trauma and in traumatized patients with or without TBI [21,22,23]. Its influence on the inflammatory processes in the brain [24] as well as remote organs upon TBI remains a subject of controversial discussion. Therefore, we evaluated whether the administration of acute ethanol will alleviate the ongoing post-TBI expression of local inflammatory mediators in trauma-distant organs, particularly in the lungs and liver.

## 2. Results

### 2.1. Ethanol Intoxication Downregulates TBI-Induced HMGB1, IL-6, and MMP9 but Enhances IL-1β Gene Expression in Liver Tissue

**Figure 1:**
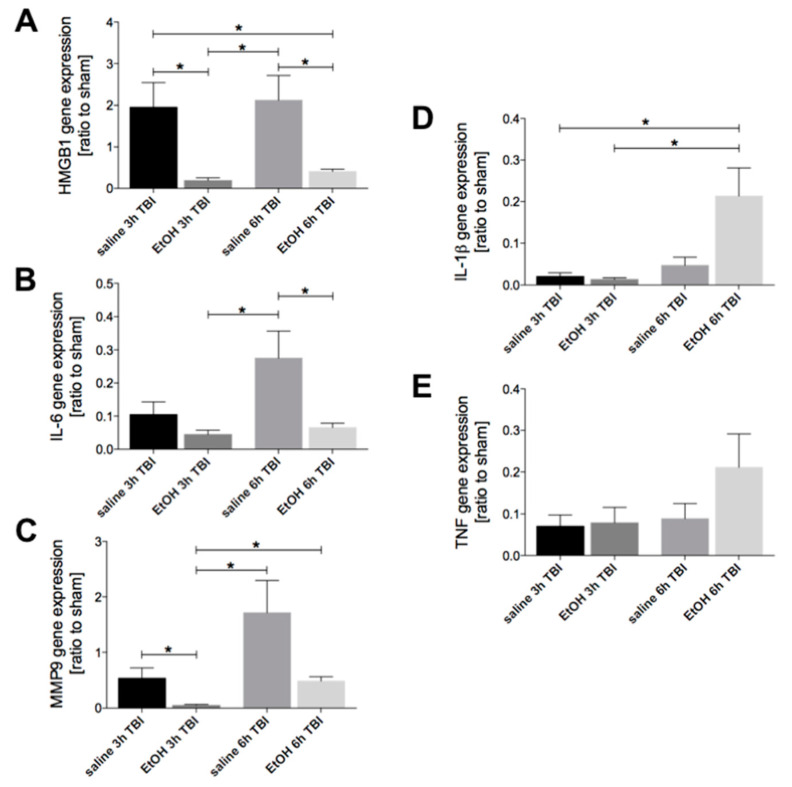
Ethanol (EtOH) intoxication downregulates traumatic brain injury (TBI)-induced HMGB1, IL-6, and MMP9 in the liver but enhances the hepatic IL-1β gene expression. Gene expression of (**A**) HMGB1, (**B**) IL-6, (**C**) MMP9, (**D**) IL-1β, and (**E**) TNF was determined. Relative mRNA expression of target genes is presented as a ratio to sham controls. Data are represented as mean ± SEM. *P* < 0.05: *: vs. indicated. The number of animals for each group is in saline sham: *n* = 6; EtOH sham: *n* = 6; saline 3h TBI: *n* =6; EtOH 3h TBI: *n* = 6; saline 6h TBI: *n* = 7; and EtOH 6h TBI: *n* = 11.

We explored the acute inflammatory response in the liver 3h and 6h after TBI by determining the expression of the proinflammatory genes HMGB1, IL-6, MMP9, IL-1β, and TNF by quantitative RT-PCR. The mRNA level of HMGB1 was significantly reduced at 3h and 6h upon TBI in the EtOH groups compared to the corresponding saline-TBI groups (*p* < 0.05, Figure 1a). The hepatic IL-6 gene expression increased markedly in the saline-treated group at 6h compared to the 3h time point, however this was not significant (Figure 1b). EtOH application significantly reduced the TBI-induced IL-6 increase at 6h compared to the corresponding saline control group (*p* < 0.05, Figure 1b). The MMP9 gene expression in the liver tissue was significantly reduced at 3h after experimental TBI in the EtOH group compared to the corresponding saline-TBI group (*p* < 0.05, Figure 1c). Interestingly, ethanol administration itself appeared to upregulate the mRNA level of MMP9 in liver tissue at 6h vs. at 3h after TBI (*p* < 0.05, Figure 1c). Although ethanol intoxication did not significantly alter the mRNA level of IL-1β in liver tissue at 3h upon TBI compared to the saline group, the gene expression of IL-1β significantly increased in the EtOH group at 6h compared to 3h upon TBI (*p* < 0.05, Figure 1d). The gene expression of IL-10 in the liver tissue was unaltered by ethanol intoxication at 3h and 6h upon TBI nor by the TBI itself (Figure 1e).

### 2.2. Ethanol Intoxication Modulates the Early Inflammatory Response in Liver Tissue upon TBI

**Figure 2:**
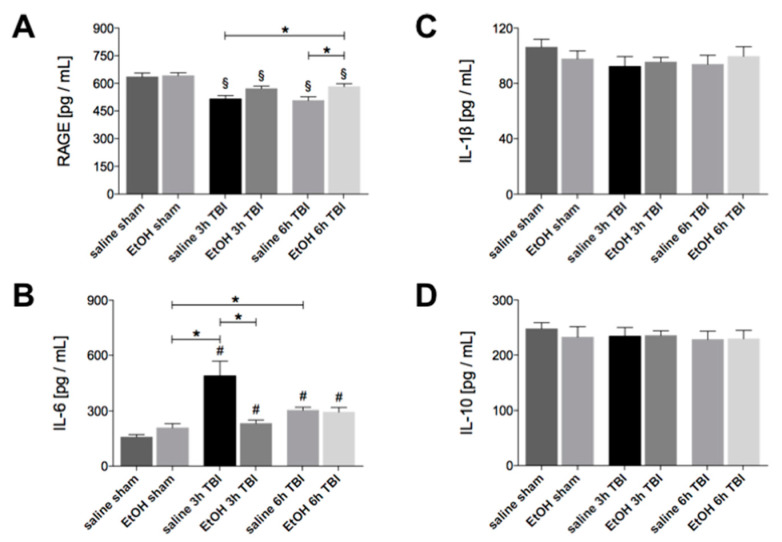
Ethanol (EtOH) intoxication modulates the early stage of inflammation in liver tissue upon TBI. Homogenate protein levels of (**A**) RAGE, (**B**) IL-6, (**C**) IL-1β and (**D**) IL-10 were determined by ELISA. Data are represented as mean ± SEM. *p* < 0.05: §: vs. both sham; #: vs. saline sham; *: vs. indicated. The number of animals for each group is in saline sham: *n* = 6; EtOH sham: *n* = 6; saline 3h TBI: *n* = 6; EtOH 3h TBI: *n* = 6; saline 6h TBI: *n* = 7; and EtOH 6h TBI: *n* = 11.

The homogenate protein expression of RAGE in liver tissue was significantly reduced at 3h and 6h upon TBI in all groups compared to both the sham groups with or without ethanol intoxication (*p* < 0.05, Figure 2a). However, the homogenate protein level of RAGE in liver tissue was significantly increased in the EtOH group at 6h after TBI compared to both saline groups after TBI (*p* < 0.05, Figure 2a). In contrast with gene expression studies, the level of IL-6 protein in liver tissue was significantly increased at 3h and 6h upon TBI in both EtOH as well as saline groups compared to both sham groups with or without ethanol intoxication (*p* < 0.05, Figure 2b). Three hours after TBI, the homogenate protein level of IL-6 in liver tissue reached a peak among all groups, which was significantly decreased by ethanol intoxication (*p* < 0.05, Figure 2b). No significant changes in the IL-1β or IL-10 protein expression at 3h and 6h upon TBI with or without ethanol intoxication were found among the groups (Figure 2c,d).

### 2.3. Ethanol Intoxication Reduces the TBI-Induced Protein HMGB1 in Liver Tissue

**Figure 3:**
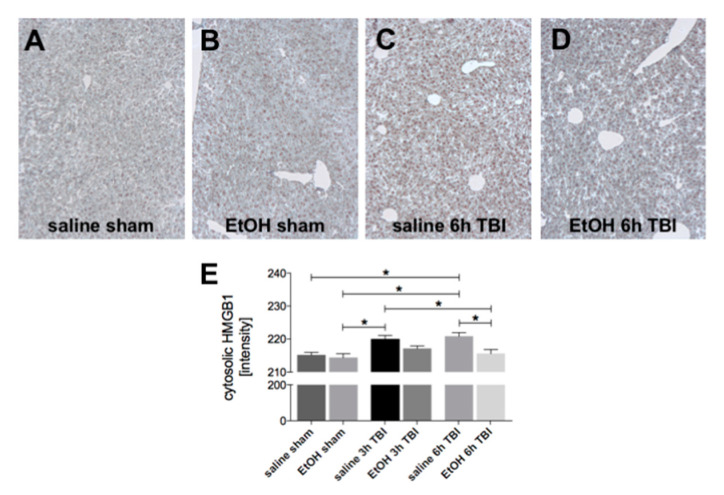
Ethanol (EtOH) intoxication reduces the TBI-induced protein HMGB1 in the liver tissue. Representative images were assessed at a 50× magnification. Immunohistological staining of HMGB1 is shown in (**A**) saline sham, (**B**) EtOH sham, (**C**) saline 6h post TBI, and (**D**) EtOH 6h post TBI. (**E**) Quantification of the intensity of protein HMGB1 staining in liver tissue after TBI in all groups. Data are represented as mean ± SEM. *p* < 0.05: *: vs. indicated. The number of animals for each group is in saline sham: *n* = 6; EtOH sham: *n* = 6; saline 3h TBI: *n* = 6; EtOH 3h TBI: *n* = 6; saline 6h TBI: *n* = 7; and EtOH 6h TBI: *n* = 11.

The expression of HMGB1 was significantly elevated in liver tissue at 3h and 6h upon TBI, as assessed by immunohistological analysis (*p* < 0.05, Figure 3a–e). Corresponding to the result of HMGB1 gene expression, ethanol intoxication significantly downregulated the TBI-induced HMGB1 presence in the liver tissue after 6h compared to the sham groups (*p* < 0.05, Figure 3a–e).

### 2.4. Ethanol Intoxication Downregulates the TBI-Induced HMGB1, IL-6, IL-1β and TNF Gene Expression in Lungs

**Figure 4:**
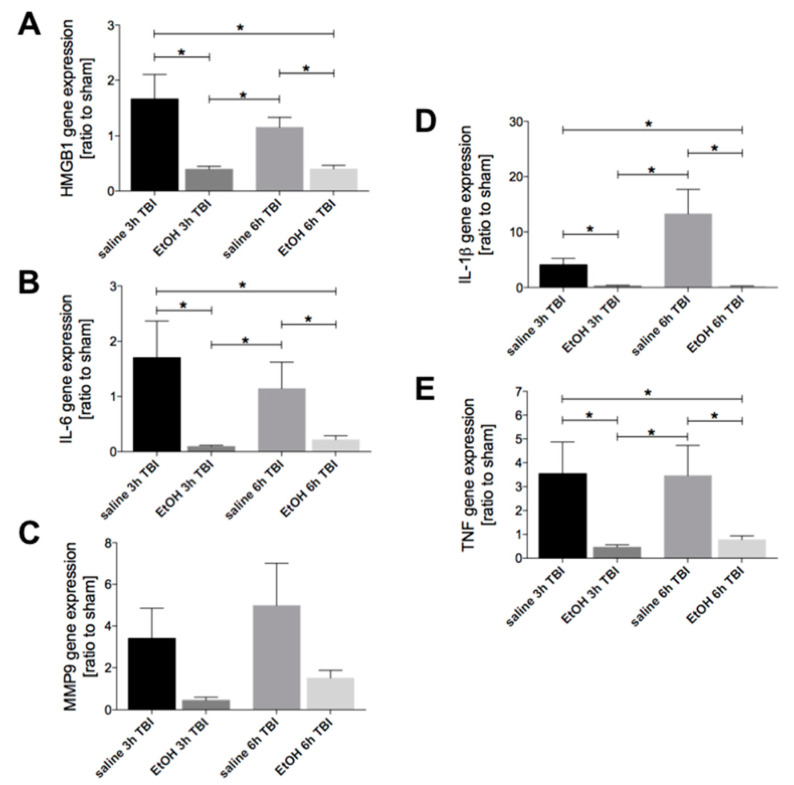
Ethanol (EtOH) intoxication downregulates the TBI-induced HMGB1, IL-6, IL-1β, and TNF gene expression in the lungs. Gene expression of (**A**) HMGB1, (**B**) IL-6, (**C**) MMP9, (**D**) IL-1β, and (**E**) TNF was determined. Relative mRNA expression of target genes is presented as a ratio to the sham controls. Data are represented as mean ± SEM. *p* < 0.05: *: vs. indicated. The number of animals for each group is in saline sham: *n* = 6; EtOH sham: *n* = 6; saline 3h TBI: *n* = 6; EtOH 3h TBI: *n* = 6; saline 6h TBI: *n* = 7; and EtOH 6h TBI: *n* = 11.

The gene expression of HMGB1 was significantly reduced at 3h and 6h in lung tissue upon TBI in EtOH groups compared to corresponding saline groups (*p* < 0.05, Figure 1a). Comparable results were found with regard to the IL-6 gene expression (*p* < 0.05, Figure 1b). There was a strong tendency toward a decrease in the MMP9 gene expression at 3h and 6h in the lungs upon EI after TBI, though it was not statistically significant (Figure 4c). The IL-1β gene expression in the lung tissue was significantly reduced at 3h and 6h upon TBI in EtOH groups compared to the corresponding saline group (*p* < 0.05, Figure 4d). Similarly, the TNF gene expression was significantly reduced at 3h and 6h in lung tissue upon TBI in EtOH groups compared to the corresponding saline control group (*p* < 0.05, Figure 4d).

### 2.5. Elevation of IL-10 In Lung Tissue Early after Experimental TBI

**Figure 5:**
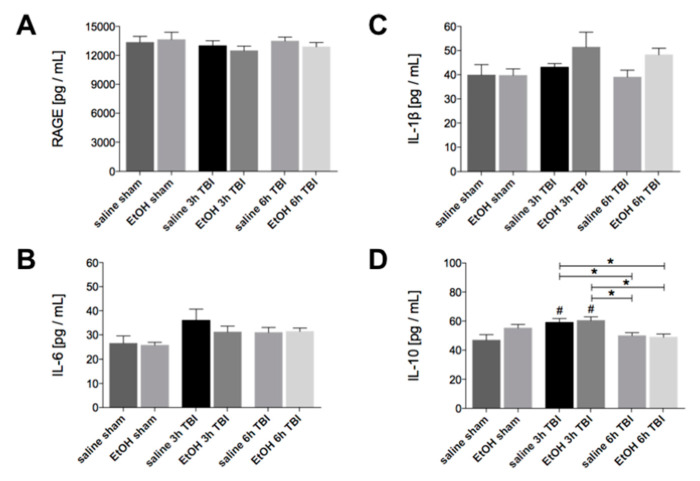
Protein expression of the anti-inflammatory cytokine IL-10 is elevated in tissue homogenates from lung tissue at the very early stage upon TBI. Levels of (**A**) RAGE, (**B**) IL-6, (**C**) IL-1β, and (**D**) IL-10 were determined in the total tissue homogenates by ELISA. Data are represented as mean ± SEM. *p* < 0.05: #: vs. saline sham; *: vs. indicated. The number of animals for each group is in saline sham: *n* = 6; EtOH sham: *n* = 6; saline 3h TBI: *n* = 6; EtOH 3h TBI: *n* = 6; saline 6h TBI: *n* = 7; and EtOH 6h TBI: *n* = 11.

Considering the potential role of the HMGB1-RAGE axis in the mechanism of lung dysfunction induced by TBI, we measured the expression of RAGE and the associated proinflammatory and anti-inflammatory cytokines [25]. Surprisingly, the homogenate protein expression of RAGE was unchanged in lung tissue at 3h or 6h upon TBI with or without ethanol intoxication (Figure 5a). Similarly, the homogenate protein expression of neither IL-6 nor IL-1β was altered by ethanol intoxication at 3h and 6h upon TBI (Figure 5b,c). However, the homogenate protein expression of the anti-inflammatory IL-10 increased significantly in lung tissue at 3h upon TBI in both the saline control as well as the EtOH group compared to the saline sham group (*p* < 0.05, Figure 5d). Interestingly, the level of IL-10 in lung tissue was significantly reduced at 6h upon TBI in the saline and EtOH groups compared to corresponding groups at 3h upon TBI (*p* < 0.05, Figure 5d).

## 3. Discussion

Ethanol intoxication (EI) inhibited the early local inflammatory response in the trauma-distant organs lungs and liver after murine TBI, as evidenced by the suppressed expression of early increased acute-phase protein IL-6 and the protein expression of HMGB1 in the liver after TBI. It was shown before that EI exerted immune-suppressive effects on neuroinflammatory and systemic responses in experimental TBI as well as in TBI patients [21,25]. Proinflammatory cytokines (e.g., TNF-α, IL-1, IL-6, and IL-8) are produced and released in LPS-activated human monocytes via the interaction between RAGE and the extracellular all-thiol form of HMGB1 in vitro [26,27]. Interestingly, blocking RAGE in an LPS-induced systemic inflammation model in rats reduced the proinflammatory cytokine levels (TNF-α, IL-1β) in both the serum and liver [28]. Considering the high RAGE protein expression in pulmonary endothelial and alveolar cells [29], a slight decrease may differ in the liver and lungs after TBI. Previously, it was shown that acute EI downregulated the parenchymal cytokine protein expression in the cerebral cortex and the gene expression in the hippocampus obtained at 3h (e.g., IL-6, IL-3, MCP-1, GM-CSF) and at 6h (e.g., IL-6, IL-2, IFN-γ) after TBI [24,25]. Consistently, in our study, gene expression was upregulated in the liver (e.g., HMGB1, IL-6, MMP9) and lungs (e.g., HMGB1, IL-6, MMP9, TNF, IL-1β) after TBI. Acute EI significantly downregulated the above-reported TBI-induced gene expression. Yet, the protein levels of IL-6 and HMGB1 were only suppressed in the liver upon EI.

HMGB1, as a common pathophysiological driver and prognostic biomarker after TBI, has the potential to constitute an innovative therapeutic target in several diseases such as neuroinflammation, epilepsy, and cognitive dysfunction [30]. Its increased production and release in cerebrospinal fluid predicted a poor outcome after pediatric TBI, and thus HMGB1 may represent a danger signal of injured cells in both local and remote organs [31]. Thermal injury in rats increased the hepatic and pulmonary HMGB1, similarly to other proinflammatory cytokines, inducing a cytokine-like effect on macrophages and monocytes [32,33]. Sub-chronic EI increased the HMGB1 level in the ipsilateral cortex at 14 days and aggravated the activation of microglia and reactive astrocytes after mild focal TBI in rats [34]. Additionally, the activation of nucleocytoplasmic translocation and secretion from the hepatocytes induced an elevated expression of HMGB1 in rodent models of alcoholic liver disease [35]. Moreover, in the brain and intestine, alcohol feeding markedly elevated the expression of proinflammatory cytokines such as HMGB1, TNF-α, IL-17, MCP-1, and IL-23 [36]. The protein expression of HMGB1 in lungs was increased at 48 h after adolescent intermittent ethanol exposure in mice. When infected with *K. pneumoniae*, the mice expressed significantly elevated inflammatory protein levels such as HMGB1, IL-6, TNF-α, RAGE, and TLR4 [37]. Interestingly, the TBI-induced HMGB1 levels were reduced by EI in the lungs and liver in our study. However, the protein expression of RAGE in the liver tissue was enhanced at 6h upon TBI by EI. Compared to the increased levels of IL-6, IL-1β, and TNF-α in other animal models, the TBI-induced distant or remote injury to lungs and liver may be less prominent upon EI due to the inhibition of the HMGB1-RAGE axis.

Severely injured trauma patients demonstrate increased systemic IL-6 levels that are also enhanced in TBI models in the liver, indicating an early stage of inflammation; however, in both models IL-6 can be reduced by acute EI [21,22]. The results are consistent with our findings confirming a TBI-induced hepatic and pulmonary increase in IL-6 gene expression which can be diminished by acute EI. However, this inhibitory effect of ethanol was not observed on the protein level in the lungs, although a slight decrease was evident at 3h post TBI. Interestingly, there is a discrepancy between the gene expression and protein expression of IL-6 at the same time point. Goodman et al. found that a transient increase in the cortical IL-6 mRNA expression was observed at 6h, while it was resolved at 24 h after TBI [38]. Additionally, the peak systemic protein concentration of IL-6 was decreased from 3h to 9h after TBI by ethanol intoxication [39]. In Figure 2b, there is no statistical difference in the protein expression of IL-6 between 3h and 6h after TBI with saline, but a slight decrease at 6h was observed. Thus, this still needs to be elucidated at further time points—e.g., 12h or 24h—in future research. In order to further evaluate the impact of EI on TBI-induced remote organ inflammation in terms of protein expression, the sample size should be increased. Our findings with reduced IL-6 gene expression upon EI and TBI are in line with previous reports showing that the LPS-induced gene expression of IL-6 was inhibited by acute EI in mice [40]. However, there are some reports that are contradictory to our findings. Teng et al. have found that acute alcohol intoxication did not modulate the inflammatory mediators (e.g., IL-6, TNF-α, IL-1, MCP-1) at 6h but restrained the resolution of inflammation at 24h after mild TBI [38]. Furthermore, Sears et al. suggested that the effects on inflammatory mediators by ethanol were tissue-dependent. They found that acute alcohol intake decreased the inflammation biomarkers (e.g., IL-2, IL-6, IL-10) in serum, while the protein levels of IL-2, IL-6, IL-1β, and macrophage inflammatory protein-1α increased upon bilateral femoral fracture with fixation in rats [41]. In chronic alcoholic patients, the effects on proinflammatory cytokines were reversed by chronic alcohol administration, which caused elevated levels of TNFα, IL-1β, and IL-6 [42]. In a contrasted acute and chronic alcohol in vitro experiment, the TLR8 or TLR4-induced TNF-α protein and mRNA expressions were decreased and the IL-10 production in human monocytes was increased by acute alcohol. However, the protein level and mRNA of TNF-α were elevated by chronic alcohol, without influencing the IL-10 production in monocytes [43].

NF-κB, as a pivotal transcriptional regulator of proinflammatory genes, promotes the production and release of proinflammatory cytokines, including IL-1, IL-6, IL-12, TNF-α, and several chemokines during inflammation [44]. The mRNA level of MMP9 and its activity were increased by activating NF-κB in TNF-α-induced monocytes [45]. Interestingly, in a rat model of hemorrhagic shock acute EI reduced the gene expression of MMP9 as well as hepatic damage early after shock via the NF-κB/MMP-9 pathway [23]. Consistently, we have obtained similar results showing that EI significantly reduced the hepatic gene expression of MMP9 at 3h upon TBI. Although a strong tendency towards a pulmonary suppression of MMP9 gene expression by EI upon TBI was observed, the effect missed statistical significance.

Interestingly, we have observed an enhanced hepatic gene expression of IL-1β at 6h after EI upon TBI. In line with this data, we previously found a transient increase of systemic IL-1β levels at 2h with unaffected and increased hepatic IL-1β gene expression at 72h after EI in rats undergoing hemorrhagic shock [23]. Given the unilateral increase in IL-1β mRNA as opposed to the hepatic protein IL-1β in our study, these results may be caused by the potential effects of ethanol on the post-translational modulation of IL-1β upon TBI. Acute ethanol exposure decreased the activation of NLRP3 inflammasome, thereby reducing the production of IL-1β and caspase-1 cleavage in neutrophils, macrophage cell line J774 and bone marrow-derived dendritic cells from mouse and human PBMCs [46,47]. However, chronic ethanol administration promoted an over-activation of NLRP3 inflammasome to increase the IL-1β secretion mediated by enhanced nitric oxide and mitochondrial reactive oxygen species in a mouse macrophage cell line (J774) [48]. Reactive acute phase proteins such as IL-1β are increased in the brain and serum after TBI [49,50]. However, a significantly reduced induction of IL-1β both on protein and mRNA levels after bacterial stimulation was observed when human blood monocytes were co-cultured with acute alcohol administration [51]. Consistently, a reduced IL-1β gene expression in the lungs upon TBI and EI was shown in our study.

Endogenous IL-10 plays an important anti-inflammatory role, reducing the lung vascular permeability, TNF-α activity in bronchoalveolar lavage fluids (BAL), neutrophil recruitment, and lung myeloperoxidase content in BAL in a lung injury model [52]. Moreover, the activity of IL-10 in BAL was enhanced by ethanol treatment at 3h post LPS-administration in mice, while this effect was suppressed after 24h by ethanol treatment [40]. However, here, the effects of EI on endogenous protein induction of IL-10 were not significant neither in the lungs nor in the liver upon TBI. This disparity may be attributed to the differences in mouse models. Interestingly, IL-10 exerted rather more protective effects regarding acute lung injury than regarding liver injury in a hemorrhagic shock model [53]. This might potentially explain the transient increase of IL-10 with its anti-inflammatory effects in the lungs differing from those observed in the liver upon TBI.

There are several limitations to our study. We have analyzed the tissue protein HMGB1, while it is known that extracellular HMGB1 mediates the activation of the immune system to produce and release inflammatory cytokines. Moreover, the timing for bio-sampling and subsequent analyses should include further time points in order to evaluate the dynamics of the investigated parameters. We performed the analyses at 3h and 6h after TBI, and thus, we were only able to solely investigate the early immune response in the trauma-distant organs lungs and liver upon TBI. Additionally, measurements of pro- and anti-inflammatory mediators in serum and BAL may provide more favorable and further important insights for the TBI-induced remote organ damage as well as the inflammatory changes dependent on EI.

In summary, our data show that during the early stage of inflammation post-TBI gene expression of inflammatory mediators is more susceptible to ethanol-induced modifications than protein levels in the lungs and liver. In our future research, we aim to investigate the inflammatory response with alcohol intake in trauma-distant organs such as the lungs, liver, intestine in the model of TBI combined with hemorrhage shock or femur fracture due to its high incidence in the emergency department. The injuries of these eventually trauma-distant organs are easily overlooked after the trauma, such as ARDS after TBI. Therefore, it is important to study the alterations in inflammatory mediators in circulation or trauma-distant local tissues. Not only the systemic inflammatory response syndrome but also the potential complications should be taken into consideration. Yet, the possible hypothesis that an acute EI could alleviate the TBI-induced inflammatory response in trauma-distant organs lung and liver potentially via inhibiting the HMGB1-RAGE axis remains to be further evaluated in future work.

## 4. Materials and Methods

### 4.1. Animals and Experimental Model

A blunt TBI model was used as previously described [54]. The experiments were approved by the local veterinary and animal experimentation service under the license n.1222. B6-SJL wild-type male mice (80–90 days) received an ethanol solution by oral gavage (20 µL/g, 5 mg/g, 32% *v*/*v* in saline) or saline as control 30 min before the TBI as previously reported [25]. The time point (30 min) was chosen for a peak blood alcohol concentration between 30 and 60 min during TBI according to the previous research [55]. After pre-medication with buprenorphin (0.1 mg/g by intraperitoneally) and sevoflurane anesthesia (4% in 96% O_2_), the scalp skin was shaved and incised on the midline to expose the skull and the animals were manually positioned in the weight-drop apparatus as previously described [54]. Then, the impactor site was localized in the center of the right parietal bone and an impactor with 333 g object falling from a height of 3 cm on the mouse skull was used to induce the TBI. The kinetic energy transferred to the skull can be calculated to be 0.098J and the impact force is approximately 32N. The mortality rate in these experiments was calculated to be slightly above 20% [24]. The injury models available can be close head injury or open head injury models [56,57]. The open head injury models include a controlled cortical impact model [58], leading to a more focal injury, which uses an electromagnetic piston to move a specific distance at a specific velocity into the brain. Another open head injury model is the fluid percussion model [59], a type of mixed injury model in which fluid pulse is injected into the epidural space. Both these models lead to tissue deformation and have therefore been avoided in the current study. Closed head injury models include different types of weight drop models. The mostly commonly used are Feeney’s weight drop model [60]; a focal injury model, the Marmarou weight drop model [61]; a model for diffused injury and the Shohami weight drop model [62]; a focal injury model. The model currently used in this paper is a modified Shohami weight drop model which also includes a Neurological Severity Score (NSS) to assess the health of the mice [54]. Subsequently, the animals received 100% O_2_, and after spontaneous breathing, the scalp skin was closed with surgical suture (Prolene 6.0). Thereafter, the animals were transferred to a single-housed recovery cage on a pre-warmed pad and ad libitum access to food and water. After 3h or 6h, the animals were sacrificed by cervical dislocation, as previously reported [24]. Then, the mice were perfused with 25 mL ice-cold PBS followed by 50 mL ice-cold 4% PFA in PBS. The liver was post-fixed for 18–24h in 4% PFA at 4 °C and embedded in OCT (Tissue-Tek). Then, 3 μm cryo-sections were cut and used.

### 4.2. Group Allocation

The animals were randomly subdivided into six groups. Sham groups received either EtOH or saline (NaCl) before beginning the experiments and underwent all surgical procedures without TBI (*n* = 6 for each group). For experimental groups, the samples were collected at 3h after TBI with saline (*n* = 6), at 3h after TBI with EtOH (*n* = 6), and at 6h after TBI with saline (*n* = 7), at 6h after TBI with EtOH (*n* = 11).

Ribonucleic acid (RNA) isolation, quantitative reverse-transcription–polymerase chain reaction (RT-PCR)

Total RNA of snap-frozen lung or liver tissues was obtained using the RNeasy-system (Qiagen, Hilden, Germany) as described in manufacturer’s manual. RNase-Free DNase Set was used to remove the remaining DNA (Qiagen). Then, the quality and quantity of the RNA were determined photometrically using the NanoDrop ND-1000 device (NanoDrop Technologies, Wilmington, DE, USA), and the obtained RNA was stored at −80 °C.

For the qRT-PCR protocol, 100 ng of each tissue sample was reversely transcribed with the Affinity script QPCR-cDNA synthesis kit (Stratagene, La Jolla, CA, USA) according to the manufacturer’s instructions. Gene-specific primers for mouse hmgb1, IL-6, IL-1beta, TNF-α, and MMP9 purchased from SABiosciences and gapdh (all, SABiosciences, SuperArray, Frederick, MD, USA) as reference gene were applied for the determination of the mRNA expression, which was performed on a Stratagene MX3005p QPCR system (Stratagene). The PCR reaction was set up with 1× RT2 SYBR Green/Rox qPCR Master mix (SABiosciences) in a 25 µL volume according to manufacturer’s protocol. Briefly, a two-step amplification protocol consisting of initial denaturation at 95 °C for 10 min, followed by 40 cycles with 15 s denaturation at 95 °C, and 60 s annealing/extension at 60 °C was applied. A melting curve analysis served to control the specificity of the gene products.

The relative expression of each target gene was calculated using the comparative threshold-cycle (CT) method (2_ΔΔCT method). Briefly, the total amount of target mRNA was normalized to the gapdh expression to provide the ΔCT and then to a calibrator consisting of samples obtained from the corresponding sham group. The relative mRNA expression of the target genes is presented as a ratio to the sham.

### 4.3. Quantification of Homogenate Protein Expression Levels by ELISA

Lung or liver tissue was snap-frozen using liquid nitrogen. For protein extraction, the tissue was homogenized in protein lysis buffer at 4 °C, and centrifuged for 30 min at 4 °C at 20,000× *g*. The supernatant protein fraction was stored at −80 °C. The protein concentrations of RAGE, IL-6, IL-1β, and IL-10 were determined using a Mouse RAGE Quantikine ELISA, and IL-6, IL-1 beta/IL-1F2, and IL-10 DuoSet ELISA kit of R&D Systems according to the manufacturer’s instructions (Wiesbaden-Nordenstadt, Germany). The Infinite M200 microplate reader (Tecan, Männedorf, Switzerland) was used to quantify the results.

### 4.4. Immunohistological Analysis of HMGB1

Cryo-frozen liver samples were sectioned and stained with anti-HMGB1 antibody. Epitope recovery was performed under steam atmosphere using R-Universal epitope recovery buffer (Aptum, Kassel, Germany) for 20 min (Retriever 2010, Prestige Medical). To block the endogenous peroxidase activity, hydrogen peroxide (Peroxidase UltraVision Block) was used. After washing with water and PBS, Anti-HMGB1 antibody (Abcam) was applied for one hour at room temperature. A secondary horseradish peroxidase-linked antibody (Histofine Simple Stain Mouse MAX PO (R), Nichirei Biosciences Inc.) and 3-amino-9-ethylcarbazol (AEC, DCS Innovative Diagnostik-Systeme, Hamburg) were used to detect specific binding. The samples were counterstained with hematoxylin. The intensity of the tissue protein HMGB1 signal was assessed using the ImageJ software in a blinded manner by an independent examiner.

### 4.5. Statistical Analysis

The differences between groups were determined by a one-way analysis of variance (ANOVA) using Kruskal–Wallis with Dunn’s post-hoc test. A p value of less than 0.05 was considered significant. Data are provided as mean ± standard error of the mean (SEM). All the statistical analyses were performed using GraphPad Prism 6 (Graphpad Software, Inc., San Diego, CA).
